# Case Report: Myocarditis After COVID-19 Vaccination – Case Series and Literature Review

**DOI:** 10.3389/fmed.2022.836620

**Published:** 2022-02-14

**Authors:** Samuel Nunn, Johannes Kersten, Marijana Tadic, Alexander Wolf, Birgid Gonska, Elina Hüll, Hanna Dietenberger, Wolfgang Rottbauer, Dominik Buckert

**Affiliations:** ^1^Department for Internal Medicine II, University of Ulm, Ulm, Germany; ^2^Department of Pathology, University of Ulm, Ulm, Germany

**Keywords:** SARS-CoV-2, myocarditis, mRNA vaccines, echocardiography, cardiovascular magnetic resonance (CMR), endomyocardial biopsy (EMB), speckle tracking

## Abstract

**Background:**

The ongoing COVID-19 pandemic demands a series of measures and, above all, the vaccination of a substantial proportion of the population. Acute myocarditis is a rare complication of the widely used mRNA-based vaccines.

**Case Presentation:**

We present a case series of four patients (three men and one woman, 16 to 47 years old) with acute pericarditis/myocarditis 3 to 17 days after mRNA vaccination. They presented with chest pain, fever, and flu-like symptoms. Diagnosis was made based on the synopsis of clinical presentation, elevated levels of troponin T and NT-proBNP, impaired systolic function on echocardiography, and findings in non-invasive tissue characterization by cardiovascular magnetic resonance imaging. Two patients also underwent endomyocardial biopsies. As none of the patients showed signs of cardiogenic shock, they were discharged from ward care only a few days after their initial presentations.

**Conclusions:**

Our data are consistent with other case reports of myocarditis early after mRNA vaccination and demonstrate the need for multimodal diagnostics. In view of its rarity and mild course, the risk–benefit ratio of vaccination remains positive compared to potential SARS-CoV-2 infection.

## Introduction

The ongoing COVID-19 pandemic is an acute medical, social, political, and economic problem (1, 2). Tremendous effort has gone into developing vaccines to prevent SARS-CoV-2 infection. To date, several mRNA-based vaccines and vaccines with adenoviruses as vectors have been approved. Since these mRNA based vaccines have not been used so broadly before, little is known about adverse events. The most common systemic adverse effects of mRNA-based vaccines are fatigue, headaches, chills, muscle pain, and fever. The initial registration studies described no cases of myocardial injury (3, 4). However, increasing evidence of myocarditis in the context of vaccination has been reported in the subsequent literature (5). This has attracted media interest due to the ongoing pandemic and has resulted in a fear of vaccination in parts of society.

Myocarditis is an inflammatory disease of the myocardium that can be caused by various infectious agents, systemic diseases, drugs, and toxins. The current guidelines of the European Society of Cardiology (ESC) also mention that non-COVID-19 vaccines can cause myocarditis (6). Myocarditis has been described to be more common in young adults and men (7). Furthermore, there is an increased risk of myocarditis within 1 week after vaccination (8). We present three cases of acute myocarditis and one case of pericarditis potentially caused by mRNA vaccines and discuss them in the context of the current literature. All patients were managed in our tertiary university care center and provided written informed consent.

## Case Reports

### Case 1

A 31-year-old woman with no preexisting diseases or cardiovascular risk factors presented in May 2021 with a shivering attack, intensifying stabbing chest and back pain, and dyspnea after moderate physical activity (NYHA II). Seventeen days previously, she had been vaccinated for the first time against COVID-19 with Comirnaty® (BioNTech/Pfizer). The following day, she registered flu-like symptoms, which quickly resolved. In the period between her vaccination and presentation, she engaged in physical activity that involved riding a bicycle for 15 km.

**Figure 1:**
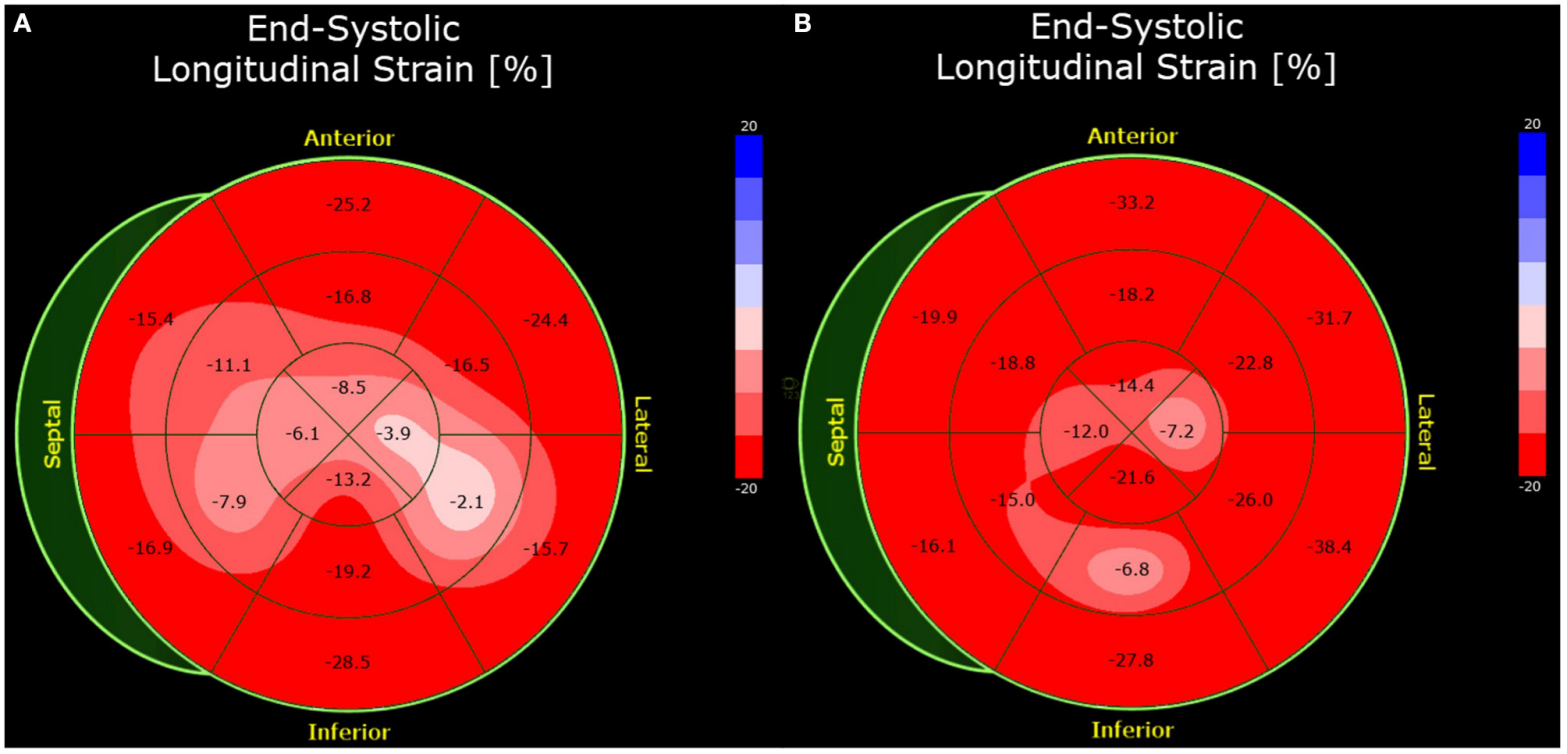
Bull's eye plot of the speckle-tracking analysis of Case 1. The global longitudinal strain was impaired by −13.4% (normal <- 18.0%) at presentation **(A)**. At 7-week follow-up, the strain analysis was normal **(B)**.

**Figure 2:**
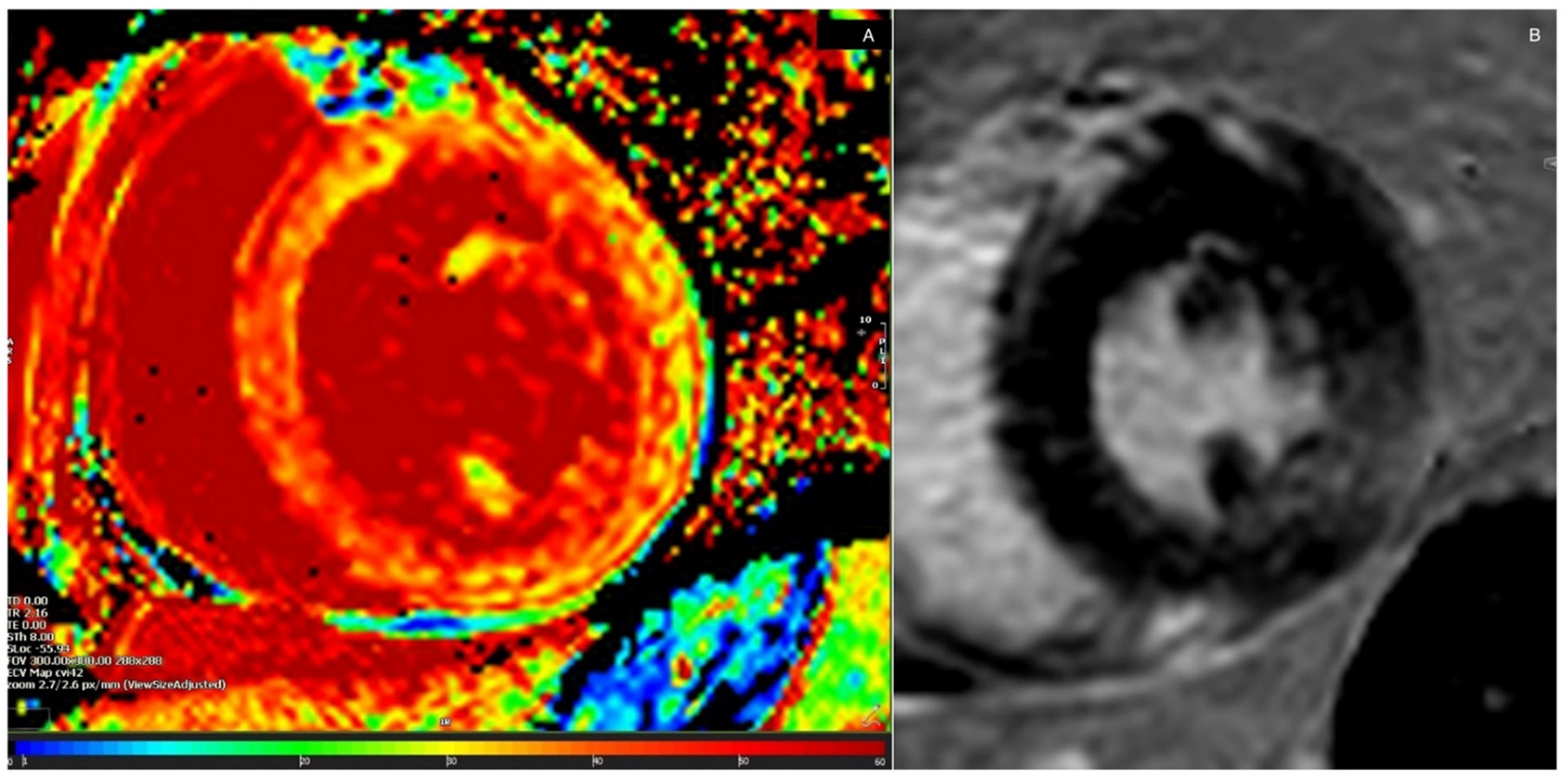
Global extracellular volume by cardiovascular magnetic resonance imaging of Case 1. Pathological values were obtained from the entire left ventricular circumference **(A)**. Focal conspicuities were shown in late gadolinium enhancement (LGE) sequences. This showed inferolateral LGE consistent with regional wall motion abnormalities on echocardiography **(B)**.

**Figure 3:**
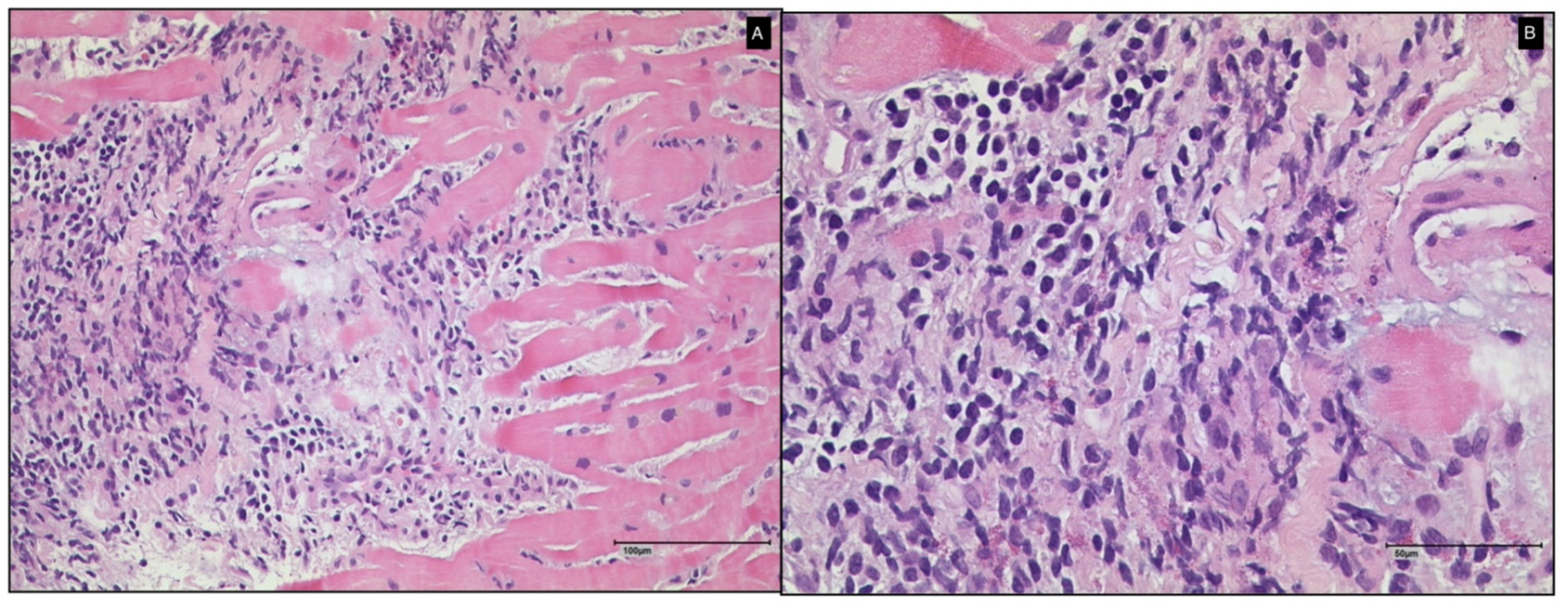
Histological findings of endomyocardial biopsy of Case 1 (**A**: 100 μm and **B**: 50 μm). A diagnosis of myocarditis without giant cells was made.

The initial physical examination showed normal blood pressure and mild tachycardia, with no signs of cardiac congestion. The initial laboratory tests showed increased levels of high-sensitivity troponin T (hsTnT) and NT-pro B-type natriuretic peptide (NT-proBNP). Transthoracic speckle-tracking echocardiography showed a mildly reduced left ventricular ejection fraction (51%) and wall motion abnormalities in the inferolateral region (Figure 1). The global left ventricular longitudinal strain was reduced to −11.0% (normal <-18.0%). Cardiovascular magnetic resonance (CMR) imaging showed increased values in parametric mapping, with a global native T1 of 1,183 ms (normal 955 ± 23 ms), T2 of 81 ms (normal <60 ms), and an extracellular volume (ECV) of 35% (normal 25.3 ± 3.5%). A subepicardial scar in the basal inferolateral region was seen in late gadolinium enhancement (LGE) sequences (Figure 2). Left heart catheterization and endomyocardial biopsy (EMB) confirmed the diagnosis of acute myocarditis (Figure 3). Polymerase chain reaction (PCR) analysis did not detect cardiotropic viruses, so there was no pathohistological evidence of a cause of myocarditis. Because of the limited ejection fraction, medical therapy with beta blocker and AT1 antagonist was initiated.

A follow-up examination after seven weeks revealed that the patient had developed movement-dependent thoracic pain but presented with no angina, dyspnea, palpitations, dizziness, or syncope. Transthoracic echocardiography showed normal left ventricular function, with no preexisting regional wall motion abnormalities. The longitudinal strain improved significantly, as shown in Figure 1. Repeat mRNA-based vaccination is not recommended.

### Case 2

A 47-year-old man with Sjogren syndrome and a history of perimyocarditis (2018) presented with a recurrence of myocarditis, with breath-dependent thoracic pressure and a fever of 38.8°C. Six days before presentation, the patient had received the second dose of Comirnaty® (BioNTech/Pfizer). The symptoms developed shortly after vaccination.

The initial presentation revealed normal blood pressure and a normal sinus rhythm. Myocardial injury was confirmed by elevated hsTnT and NT-proBNP levels. An echocardiographic examination detected no regional wall motion abnormalities. Pericarditis was confirmed by CMR because of LGE in the basal and midventricular pericardium. No LGE was detected in the myocardium. Furthermore, T1 and T2 mapping and feature-tracking strain analysis showed normal values, which is why EMB was not performed. Consistent with Sjogren's syndrome, Ro-52, SSA and SSB parameters are elevated.

### Case 3

A 16-year-old male with a family history of myocardial infarction presented with a fever and head, limb, and chest pain after receiving the second dose of Comirnaty® (BioNTech/Pfizer) 3 days previously. He reported self-medication with ibuprofen, which had improved his symptoms.

**Figure 4:**
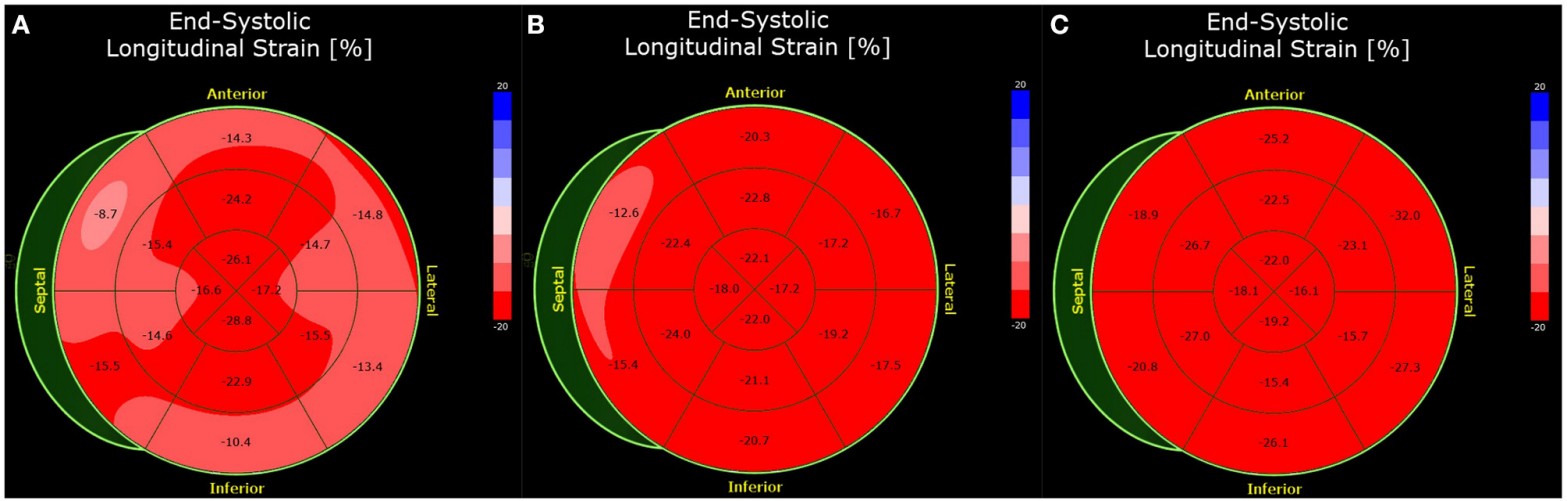
Bull's eye plot of the speckle-tracking analysis of Case 3. The global longitudinal strain was impaired by −17.1% (normal <-18.0%) at presentation **(A)**. Before discharge 4 days later, the strain analysis is improved (-19.1%) **(B)**. At 3 months follow-up, the strain analysis was normal (-21.8%) **(C)**.

As in the other three cases, there was no indication of cardiogenic shock or congestion. The patient had the highest hsTnT values of all described cases, with a maximum of 1,361 ng/ml upon admission (normal < 14 ng/ml). His heart rate was normal, with elevations in the inferior and anterior leads (II, III, aVF, V3–V6) on an initial 12-lead electrocardiogram (ECG). Echo showed a midrange reduction in the left ventricular ejection fraction. Due to the ECG changes, left heart catheterization and EMB were performed soon after admission. No evidence of coronary artery disease was found. EMB showed fibrosis but no clear evidence of myocarditis. Moreover, there were no giant cells or signs of amyloidosis. Testing for viruses by PCR is unremarkable, so that no viral genesis of the myocarditis can be assumed. Accordingly, CMR showed a subepicardial focal scar in the basal anteroseptal region in LGE. T2 and ECV were elevated in the anteroseptal region (T2: 63 ms; ECV: 32%). The patient was hospitalized and observed for 4 days. In addition, a strain analysis was performed at the beginning and end of the inpatient stay, which showed a significant improvement of the global longitudinal strain within the few days (Figure 4).

Three months later, a follow-up examination was performed. The patient had good cardiopulmonary exercise capacity without angina, dyspnea, syncope, dizziness or palpitations. Echocardiography showed normalized ejection fraction, so heart failure therapy was discontinued. Speckle tracking analysis showed a fully recovered longitudinal strain compared with the two echocardiographic studies previously (Figure 4). Based on the findings and a period of 3 months after the acute event, there is no reason for further abstinence from sports.

### Case 4

A 24-year-old man who had received the second dose of the Moderna COVID-19 vaccine 4 days previously initially presented to a general practitioner, who referred him to the hospital with suspected myocarditis because of a high fever (40.5°C) and non-significant elevations on a lead V2–V4 ECG. He reported retrosternal pain in association with deep inspiration, as well as a sore throat and cough. Self-medication with ibuprofen provided no relief.

Elevated hsTnT and NT-proBNP levels indicated myocardial injury. The patient's blood pressure and heart rate were normal. An ECG showed a sinus rhythm with preexisting ST changes. CMR showed normal left ventricular function with LGE in the basal and inferior pericardium. These findings suggested acute perimyocarditis. In the presence of normal left ventricular ejection fraction and absent LGE, EMB was not performed because of lack of therapeutic consequence. The patient was discharged 2 days later.

An overview of the laboratory and imaging data of all four patients is presented in Table 1.

**Table 1:** Overview of clinical, laboratory, and imaging data of the four cases.

**Characteristic**	**Case 1**	**Case 2**	**Case 3**	**Case 4**
Age	31	47	16	24
Sex	Female	Male	Male	Male
Vaccine	1st dose of BioNTech/Pfizer	2nd dose of BioNTech/Pfizer	2nd dose of BioNTech/Pfizer	2nd dose of Moderna
Time from vaccination to admission (days)	17	6	3	4
Relevant preexisting conditions	–	Sjogren syndrome, perimyocarditis (2018)	Family disposition	
Time from admission to discharge (days)	4	7	4	2
**Biomarkers**				
**hsTnT (ng/l, normal** **<** **14)**				
First admission	223	43	1,361	412
Peak value	549	202	2,170	412
**NT-proBNP (pg/ml, normal** **<** **130)**				
First admission	2325.0	579.0	1245.0	550.0
Peak value	2325.0	579.0	1245.0	550.0
**CRP (mg/l, normal** **<** **5)**				
First admission	12.0	97.8	7.1	52.0
Peak value	12.0	97.8	43.5	52.0
**Left and right ventricular volumetry by cardiovascular magnetic resonance imaging**				
LVEDV (ml)	155	178	180	156
LVEDVI (ml/m^2^)	80	77	89	78
LVEF (%)	52	53	50	69
SV (ml)	81	92	90	108
RVEDV (ml)	128	194	185	154
RVEDVI (ml/m^2^)	66	84	92	77
RVEF (%)	70	53	46	64
**Tissue characterization by cardiovascular magnetic resonance imaging**				
LGE	Basal inferolateral subepicardial	Basal and mid-ventricular pericardium	Basal anteroseptal subepicardial	Basal anterior and inferior pericardium
Native T1 (ms, normal 955 ± 23)	1,183	970	1,107	992
T2 (ms, normal < 60)	81	53	• 61 (anterolateral) • 63 (anteroseptal)	50
ECV (%, normal 25.3 ± 3.5)	35	27	• 28 (basal) 31 (anterolateral) 32 (anteroseptal)	26

*hsTNT, high sensitivity troponin T; NT-proBNP, NT-pro B-type natriuretic peptide; CRP, C-reactive protein; LVEDV, left ventricular end diastolic volume; LVEDVI, left ventricular end diastolic volume index; LVEF, left ventricular ejection fraction; SV, stroke volume; RVEDV, right ventricular end diastolic volume; RVEDVI, right ventricular end diastolic volume index; RVEF, right ventricular ejection fraction; LGE, late gadolinium enhancement; ECV, extracellular volume*.

## Discussion and Literature Review

Myocarditis has already been identified as an adverse event of mRNA vaccines. There were justified clinical suspicions that the presented myocarditis cases were caused by vaccination, although a causal relationship could not be fully established in all cases. Case 2 presented a challenge in the differential diagnosis between myocarditis and preexisting rheumatic disease. We included this case to show that underlying conditions may also induce cardiac involvement.

The diagnosis of myocarditis is challenging regardless of the causative agent and should be performed by an experienced clinician based on a synopsis of symptoms, laboratory data, imaging results, and histopathological findings (9–11). The clinical symptoms are broad with low specificity and include chest pain, acute or subacute shortness of breath, acute or chronic heart failure, palpitations, arrhythmia, and unexplained cardiogenic shock (11). There is no specific blood test, but biomarkers of cardiac injury such as troponin or NT-proBNP can be elevated (6, 11, 12). The ESC recommends that in all patients with clinically suspected myocarditis should be considered for selective coronary angiography and EMB (6). It is used to confirm the diagnosis of myocarditis and to identify the underlying etiology. Furthermore, the analysis provides key information on the treatment strategy and prognosis (6, 11–13). The most scientific statements about EMB are based on the classical histopathological Dallas criteria, which do not include methods established after that time such as immunohistochemistry or viral genome analysis (10, 14). EMB has a high false negative rate due to its susceptibility to sampling errors (15, 16). The AHA and ESC statements are based on consensus recommendations in the absence of large clinical trials to clarify the role of EMB for further management and cause-specific therapy (13). Therefore, EMB was not performed in cases 2 and 4 in the presence of normal ejection fraction and absence of LGE. In Case 3, this was also a potential cause of the negative EMB in the presence of otherwise undoubtful diagnostics. EMB is recommended in the case of a new onset of a reduced ejection fraction within 2 weeks and hemodynamic compromise, such as cardiogenic shock (17).

To describe the myocardial deformation, echo strain analysis based on speckle tracking is a reliable and feasible tool (18). Deformation imaging is useful to detect subclinical systolic or diastolic functional impairment (19). Left ventricular strain and strain rate analyses appears to be a good prognostic tool in patients with reduced and normal left ventricular ejection fraction (20). With normalized left ventricular ejection fraction and strain measurement at follow-up as described in Case 1, a repeat MRI was not performed because there was no therapeutic consequence. Furthermore, it could be shown in case 3 that the speckle tracking analysis can describe an improvement of global longitudinal strain within a few days indicating good short term prognosis.

CMR is also widely used due to its safety and plays a key role in the diagnosis of inflammatory myocardial diseases. Optimal results are obtained by combining a T1-based criterion (LGE, native T1, or ECV) with a T2-based criterion (Updated Lake Louise Criteria) (21, 22). Although CMR characterizes the tissue, EMB cannot be replaced (6). CMR guidance to enhance the diagnostic accuracy of EMB is possible but should not delay EMB in life-threatening presentations (6, 10).

A central role of the therapy of myocarditis is the optimal treatment of possible arrhythmias as well as heart failure. In hemodynamically stable patients, the classic agents for the treatment of heart failure are used (6, 23). In pericardial involvement, NSAIDs should be used and colchicine is also recommended as an adjunct to aspirin and NSAID therapy (23, 24). In the described cases, medical therapy for heart failure was initiated. In addition, following the AHA and ESC guidelines, abstinence from sports was recommended for 6 months or until follow-up (6, 13). The guidelines make clear that the few studies primarily address competitive sports, but the Task Force of the ESC believes that the recommendations should also be applied as expert opinion to amateur sports (6).

In the case of myocarditis due to mRNA vaccination, repeat mRNA-based vaccination was not recommended to our patients, although no study data are available until now. As shown in Case 1, vaccination with the Johnson & Johnson vaccine was performed without complications, so that the switch to a vector-based vaccine seems reasonable. A third vaccination in myocarditis due to mRNA vaccine should be performed as well with a different vaccination technology such as vector vaccines.

The most common symptoms of COVID-19 are fever, cough, and dyspnea, which are also known symptoms of pneumonia (2). The virus can enter human cells that express ACE-2, such as those in the lungs, heart, and renal and gastrointestinal tracts, with the help of its spike protein. A “cytokine storm” occurring after 7 to 14 days can lead to severe disease (25, 26). The probability of severe disease depends, among other factors, on comorbidities. Given that a cytokine storm may play a key role in the severity of the disease, the question that arises is whether it can also be triggered by mRNA vaccination.

The Israeli Ministry of Health initiated an active surveillance program for 6 month from December 2020 through May 2021 to monitor the adverse events of COVID-19 vaccines. Among 5.1 million fully vaccinated individuals, 136 were diagnosed with myocarditis. Most (95%) cases had a mild course. Compared with the pre-pandemic incidence of myocarditis obtained from the Israel National Hospital Discharge Database from 2017 to 2019, the second dose of mRNA vaccination resulted in a standardized incidence ratio of 5.3 (27). Young males were the most likely to suffer this adverse event in the first week after receiving the second dose (27). These data are in line with the myocarditis frequency of 1 to 17,000 after the second dose reported by the vaccination committee of the German Robert Koch Institute in August 2021 (28). Cohort studies in China have reported rates of myocardial injury, such as myocarditis, ranging from 7 to 17% among hospitalized COVID-19 patients. The percentage rose to 22% for patients requiring intensive care and to 59% for deceased patients (29). In a case series of 150 patients, 7% of the 68 deaths were due to myocarditis with subsequent circulatory failure. Another 22 deaths (33%) were associated with myocarditis and respiratory failure. Consequently, a definite cause of death could not be determined (30). Conversely, no cases of fatal myocarditis have been reported in the context of mRNA vaccination. Barda et al. (31) reported that vaccination increased the risk of myocarditis by a factor of 3.2. The risk difference calculated by 100,000 persons is 2.7 (CI 1.0 to 4.6). To put this risk in context, 240,000 SARS-CoV-2 infections were studied to compare the incidences of the same complications. This showed an 11.0 myocarditis per 100,000 persons (31). Data from the Premier Healthcare Database Special COVID-19 Release, which is a large US hospital-based database, showed an overall adjusted myocarditis risk ratio of 15.7 in COVID-19 patients. The risk difference is higher in male than female. The highest risk ratio in terms of age was in the group under 16. The second peak was reached after a reduction until the age of 40 with a subsequent increase in the group of patients over 75 years (risk ratio 31.6, CI 25.9–37.2) (32). Therefore, the question arises as to how the risk ratio regarding myocarditis relates between infection and vaccination in young people or children. A preliminary publication by Singer et al. examined that in the highest risk group, consisting of adolescents between 12 and 17 years old, the risk of myocarditis was 5.9-fold higher with infection compared with mRNA vaccination (33). In line with this, a British study of more than 38 million vaccinated persons shows that although the risk of myocarditis increases after vaccination, this is significantly lower compared with myocarditis after SARS-CoV-2 infection (1–10 vs. 40 extra events per 1,000,000 persons) (8). It is not yet known whether abstaining from intense physical activity for a few days after vaccination can reduce the risk of myocardial involvement, as has been recommended in myocarditis of other causes (6). Fear of this cause of myocarditis may have led to vaccine hesitancy in parts of society, which has been stoked by some media. In view of the ongoing pandemic and this rather rare adverse side effect, which mostly shows a mild clinical course, this should not be supported for rational reasons.

## Conclusion

Myocarditis may be an exceptionally rare complication after vaccination against SARS-CoV-2. The clinical course of the cases described herein was mild. The performed diagnostics conformed to current guidelines and substantiated the suspicion of myocardial involvement. Based on the currently available knowledge, the benefits of vaccination outweigh its potential risks. Therefore, broad vaccination is recommended. Nevertheless, we recommend further investigation into the adverse effects of the new mRNA vaccine technology, which may be used for most vaccines in the future.

## Data Availability Statement

The original contributions presented in the study are included in the article/supplementary material, further inquiries can be directed to the corresponding author/s.

## Ethics Statement

Written informed consent was obtained from the individual(s) for the publication of any potentially identifiable images or data included in this article.

## Author Contributions

SN and JK: concept and writing of the manuscript. MT, AW, BG, and EH: interpretation of the sources and patient acquisition. MT and HD: image example. WR and DB: supervision and concept. Each author has read and approved the final draft. All authors contributed to the article and approved the submitted version.

## Conflict of Interest

The authors declare that the research was conducted in the absence of any commercial or financial relationships that could be construed as a potential conflict of interest.

## Publisher's Note

All claims expressed in this article are solely those of the authors and do not necessarily represent those of their affiliated organizations, or those of the publisher, the editors and the reviewers. Any product that may be evaluated in this article, or claim that may be made by its manufacturer, is not guaranteed or endorsed by the publisher.
